# Supplementary material for: Endogenously Produced Jasmonates Affect Leaf Growth and Improve Osmotic Stress Tolerance in Emmer Wheat
Source: Biomolecules. 2023 Dec 12;13(12):1775. doi: 10.3390/biom13121775 (PMC10742046; doi:10.3390/biom13121775)
Supplement: Supplementary file 1 [file biomolecules-13-01775-s001.zip › biomolecules-2689752-supplementary.pdf]

Supplementary Material

Endogenously produced jasmonates affect leaf growth and improve osmotic stress tolerance in emmer wheat

Table S1 Segregation analysis of *GFP* expression in T_1 progeny of self-pollinated primary transgenic plants T_0 of emmer wheat 'Runo' transformed with pAOS-bar

T_0 plant	Number of T_1 seeds tested	Seedlings T_1			
		Observed GFP segregation ratio (positive:negative)	Ratio	χ^2 value for the expected segregation ratio (positive:negative) *	
				3:1	15:1
RA1	141	14:127	0.1:1	318.41	1690.72
RA2	184	125:59	2.1:1	4.90	209.28
RA3	147	110:37	3.0:1	0.00	89.81
RA4	267	206:61	3.4:1	0.66	125.51
RA5	124	08:106	0.2:1	241.94	1328.59
RA6	No seeds formed				
RA7	277	198:79	2.5:1	1.83	234.46
RA8	10	6:4	1.5:1	1.20	19.44
RA9	257	243:14	17.4:1	52.40	0.28
RA10	63	0:63	-	-	-

*if χ^2 value is above 3.84 ($p > 0.05$) the observed segregation ratio is significantly different from the expected ratio

- plants with *AtAOS* expression according RT-PCR

- plants displaying segregation of transgenes in correspondence with Mendelian 3:1 or 15:1 ratios

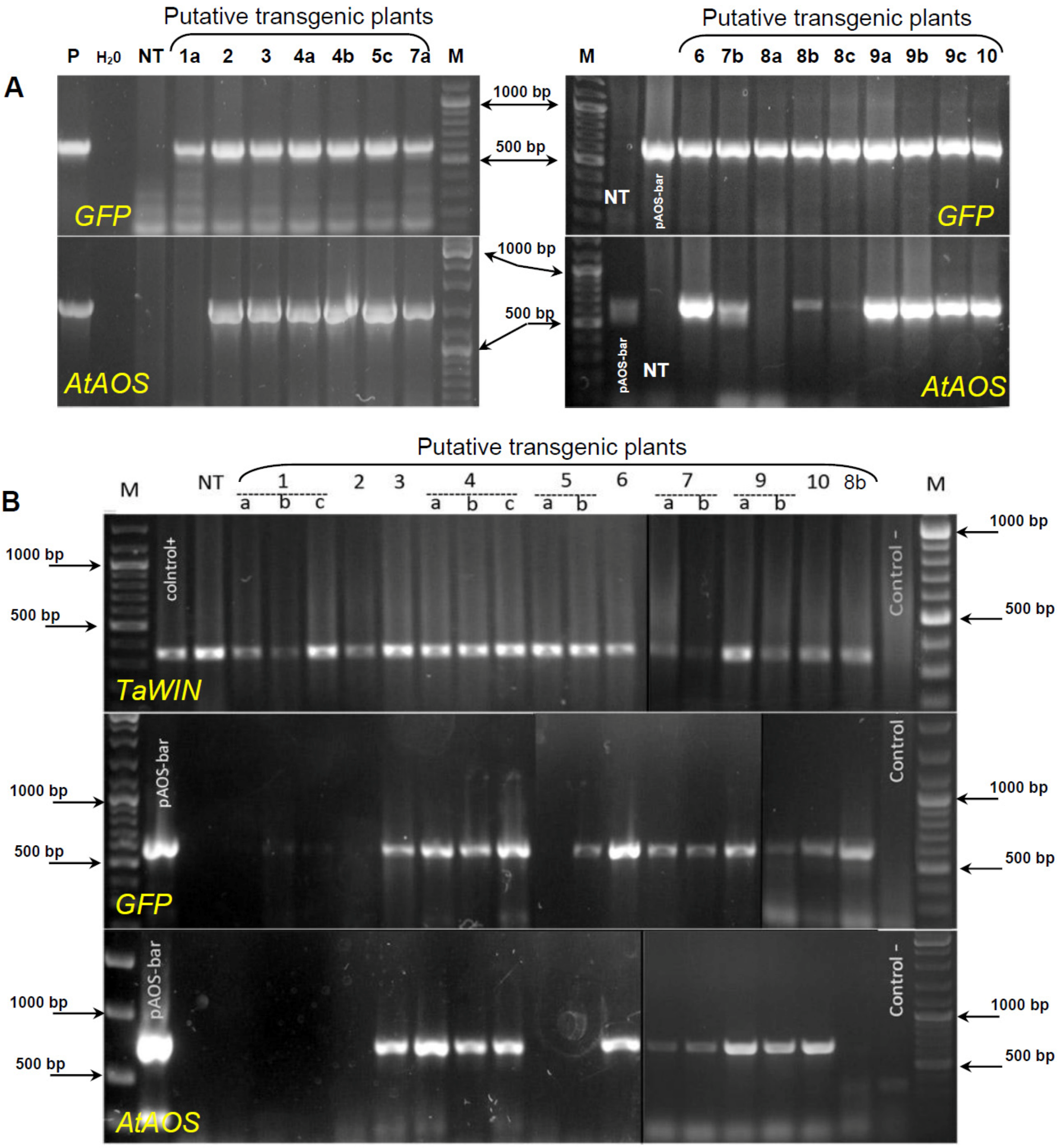

Figure S1. Analysis of putative transgenic plants of emmer wheat Runo (*T. dicoccum*) for integration and expression of transgenes from pAOS-bar vector.
(**A**), PCR analysis for the integration of sequences encoding *GFP* (top panel) and *AtAOS* (bottom panel) genes into genome of putative transgenic plants of emmer wheat. (**B**), End-point RT-PCR analysis on the total RNA of T_0 plants (at heading stage) for the expression of the reference gene *TaWIN1* (top panel), *GFP* gene (middle panel) and *AtOPR3* (bottom panel). Lane M, DNA ladder as a molecular weight marker; Lane P, plasmid DNA pAOS-bar; Lane NT, non-transgenic wheat plant Runo, Lanes labelled 1–10 represent putative transgenic wheat plants established in greenhouse; in some cases, two to three plantlets were regenerated from one explant, and plantlets were independently analysed for transgenes insertion and expression (indicated as *a,* or *b,* or *c*).

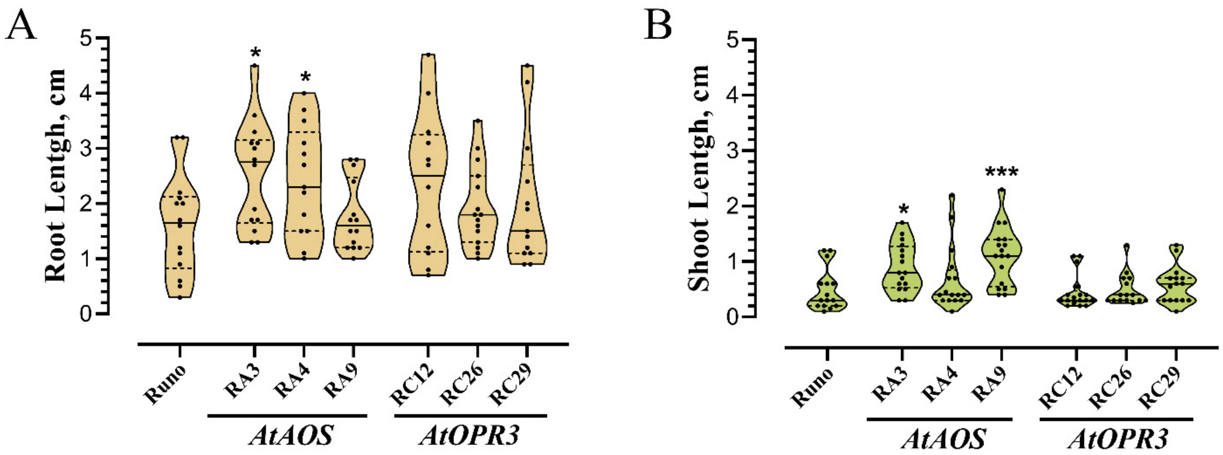

Figure S2. The growth rate of transgenic plants' seedlings overexpressing *AtAOS* (RA3, RA4, and RA9) and *AtOPR3* (RC12, RC26, and RC29) and non-transgenic plants (Runo) under osmotic stress conditions – Experiment 2. Violin plots display the length of the longest roots (yellow color, A) and coleoptiles (green color, B) grown on 20% PEG 6000. Each violin represents data from 14-20 seedlings; the solid horizontal lines within the violin show the median values, the dotted lines depict the 75th and 25th percentile of the distribution, dots are the values of individual measurements. Stars indicate statistically significant difference between genotypes determined by one-way analysis of variance (ANOVA) followed by Tukey's post hoc test ("*", $p \leq 0.05$), ("***", $p \leq 0.001$).

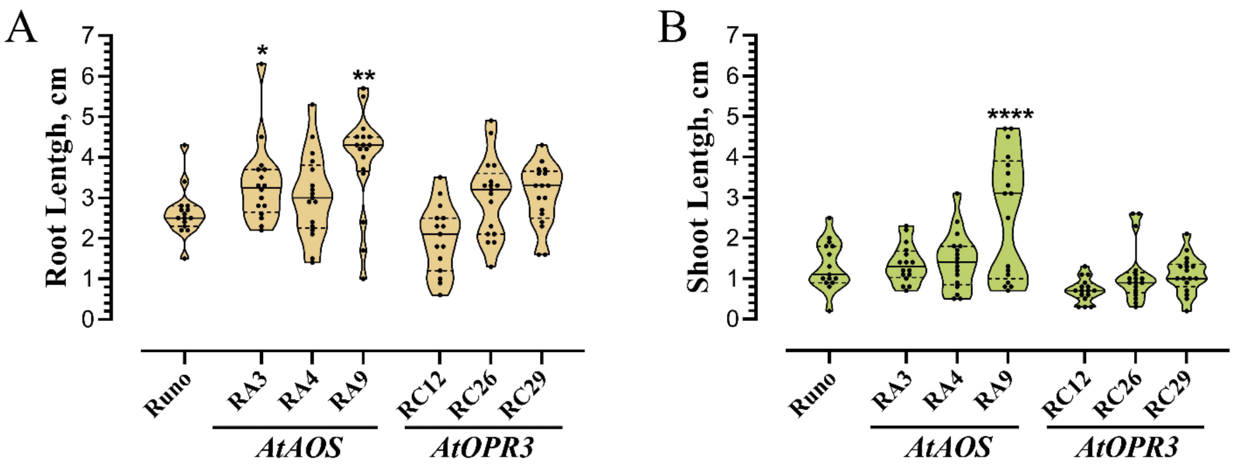

Figure S3. The growth rate of transgenic plants' seedlings overexpressing *AtAOS* (RA3, RA4, and RA9) and *AtOPR3* (RC12, RC26, and RC29) and non-transgenic plants (Runo) under osmotic stress conditions – Experiment 3. Violin plots display the length of the longest roots (yellow color, A) and coleoptiles (green color, B) grown on 20% PEG 6000. Each violin represents data from 14-20 seedlings; the solid horizontal lines within the violin show the median values, the dotted lines depict the 75th and 25th percentile of the distribution, dots are the values of individual measurements. Stars indicate statistically significant difference between genotypes ("*", $p \leq 0.05$), ("**", $p \leq 0.01$), ("****", $p \leq 0.001$).

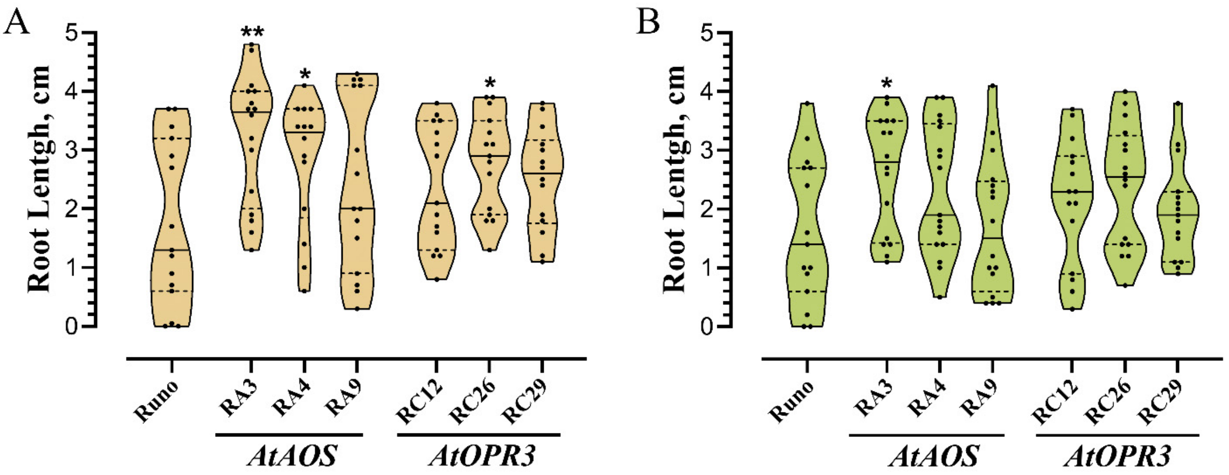

Figure S4. The growth of seedlings of transgenic plants overexpressing *AtAOS* (RA3, RA4 and RA9) and *AtOPR3* (RC12, RC26, and RC29) and non-transgenic plants Runo under osmotic stress conditions (when freshly collected seeds used). Violin plots display the length of the longest roots (yellow color, A) and coleoptiles (green color, B) after 21 days of incubation on 25% PEG 6000 followed by the 4 days of incubation after stress was removed by dilution of PEG solution with water. Each split violin represents data from 17-25 seedlings; the solid horizontal lines within the split violin shows the median values, the dotted lines depict the 75th and 25th percentile of the distribution, dots are the values of individual measurements. Stars indicate statistically significant difference between genotypes (“*”, $p \leq 0.05$), (“**”, $p \leq 0.01$).
